# Announcement of the 2016 *Polymers* Young Investigator Award

**DOI:** 10.3390/polym8030065

**Published:** 2016-02-26

**Authors:** Alexander Böker

**Affiliations:** Fraunhofer-Institut für Angewandte Polymerforschung, Lehrstuhl für Polymermaterialien und Polymertechnologie, Universität Potsdam, Geiselbergstraße 69, 14476 Potsdam-Golm, Germany; boker@mdpi.com

Dear readers of *Polymers*,

Finally, after an extensive voting period, we are proud to present the first winner of the *Polymers* Young Investigator Award to:

**Figure:**
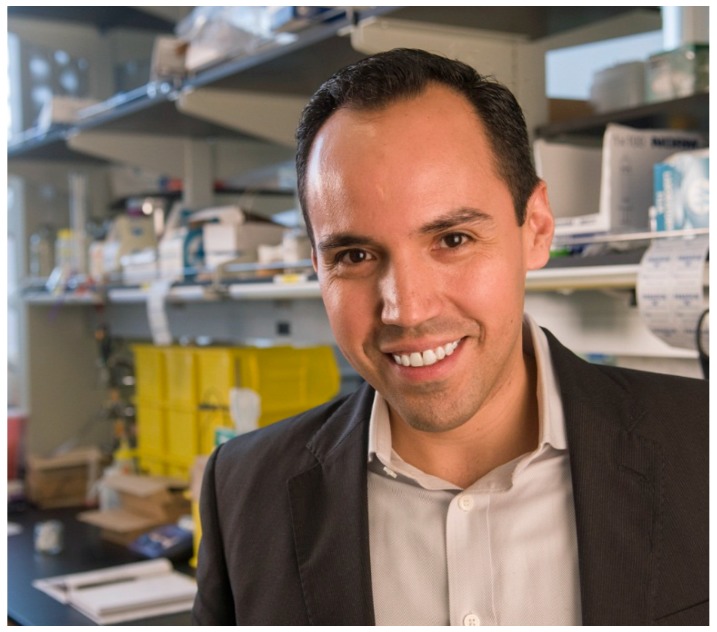
**Dr. Luis M. Campos**

who is an assistant professor at the Chemistry Department of Columbia University, USA. He was selected by the evaluation committee of *Polymers* Investigator Award from 38 candidates who were proposed by at least two colleagues in their field of expertise. Fifteen of the candidates are working in the United States, 13 in Europe and 10 at universities in Asian countries. The proposed candidates represented a diverse range of fields in polymer science. Dr. Campos received his Ph.D. with Professor Miguel A. Garcia-Garibay and Professor Kendall N. Houk at University of California, Los Angeles (UCLA) in 2016 and did his *postdoc* training at the University of California, Santa Barbara (UCSB) under the supervision of Craig J. Hawker. At the age of 37, Dr. Campos has already achieved an extraordinary standing in the polymers community. His excellent work focuses on the design and application of polymeric materials, for example, solar cells and organic light emitting diodes; all topics of high societal and economic impact. His research has been featured in highly ranked journals such as the *Nature* family, *Angewandte Chemie*, and *Journal of the American Chemical Society (JACS),* to name a few. To date, he has co-authored over 60 articles and has received numerous awards, including the American Chemical Society (ACS) Arthur C. Cope Scholar Award, The Office of Naval Research (ONR) Young Investigator Award, The National Science Foundation (NSF) CAREER Award, 3M Non-Tenured Faculty Award, Cottrell Scholar Award, The Inter-American Photochemical Society (I-APS) Young Faculty Award, the *Journal of Physical Organic Chemistry* Award for Early Excellence. This finally led to invitations to numerous prominent lectures all over the world. Moreover, his work constitutes not only high level basic research, but has proven to be highly relevant in industry which is reflected in 10 patents filed by him and his coworkers.

In addition to the cash prize and plaque, Dr. Campos will be an invited speaker at the 2018 *Polymers* conference.

On behalf of the *Polymers* Editorial office staff and editorial board members, I wish to congratulate Dr. Campos on his excellent performance and wish him all the best for his future career.

